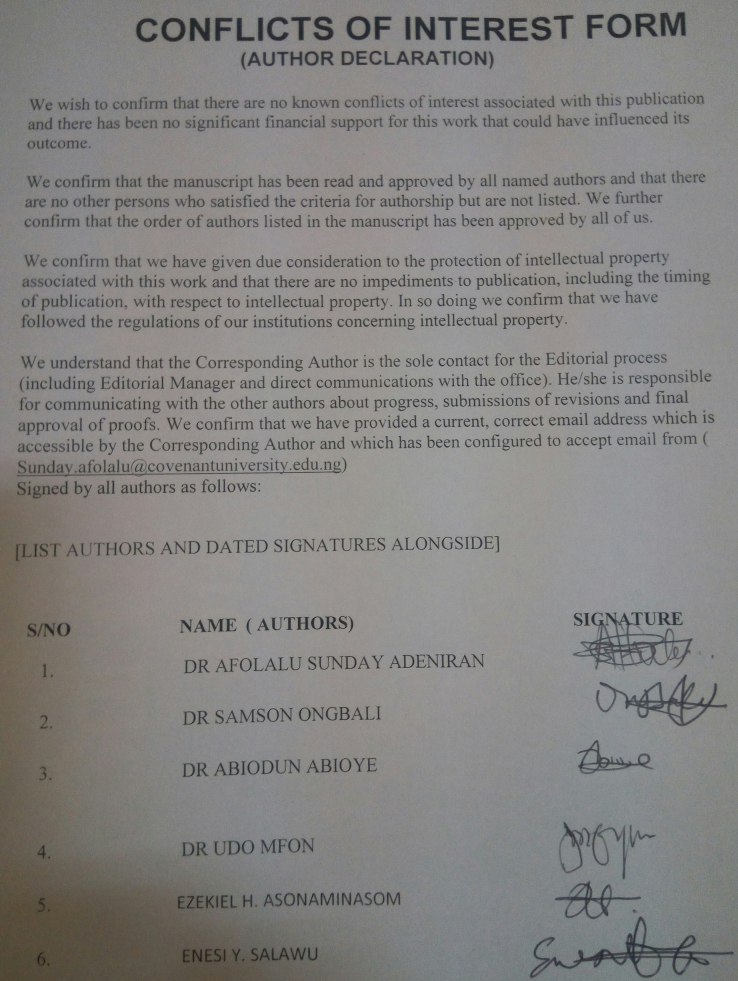# Dataset on experimental investigation of optimum carburizing temperature and holding time of bi- nano additives treatment of AISI 5130 steel

**DOI:** 10.1016/j.dib.2018.07.025

**Published:** 2018-07-19

**Authors:** Sunday A. Afolalu, Ezekiel H. Asonaminasom, Samson O. Ongbali, Abiodun A. Abioye, Mfon O. Udo, Enesi Y. Salawu

**Affiliations:** Mechanical Engineering Department, Covenant University, Ota, Ogun State, Nigeria

**Keywords:** Bi-nano additive, AISI 5130 steel, Carburizing temperature, Time, Optimum

## Abstract

Investigation of optimum carburizing temperature and holding time on bi- nano additives treatment of AISI 5130 steel was presented in this study. AISI 5130 steel of 100 kg mass of 0.35% carbon content was buried in pulverized additives consisting of palm kernel and coconut shell using eggshell as an energizer. Four sets of 150×150×150 mm^3^ steel boxes packed with additives mixed at varying weight ratio of 50: 30:20 and sixty-four pieces of 20×20×5 mm^3^ AISI 5130 steel were case hardened using muffle furnace (2500 °C max capacity) at respective temperatures and time of 950, 1000, 1050, 1100 °C and 60, 90, 120, 180 min. The core, interface and surface hardness of the treated samples with their respective weight loss, wear volume and rate were investigated. This dataset could be used in nano-composite match mixed ratio and optimization of carburizing medium and time for any industrial used case hardened steel.

**Specifications Table**

**Table:** 

Subject area	Mechanical Engineering and Surface Engineering.
More specific subject area	Production Engineering and Materials Engineering.
Type of data	Table, text and graph.
How data was acquired	Elemental composition of the steel was checked using spectrometer 675×321. Hardness tester (Brinell with 3000 kg test force and 10-mm diameter carbide) was used to analyze the surface, interface and core hardness of the treated samples. The weight loss, wear volume and rate were checked using Rotop-V machine.
Data format	Raw and analyzed.
Experimental factors	Four sets of 150×150×150 mm^3^ steel boxes packed with additives mixed at varying proportion ratio of 50:30:20 and sixty four pieces of 20×20×5 mm^3^ AISI 5130 steel were used.
Experimental features	AISI 5130 steel of 100 kg mass with 0.35% carbon content was buried in pulverized additives consisting of palm kernel and coconut shell using eggshell as an energizer. Four set of steel boxes packed with additives mixed at varying proportion ratio of 50:30:20 and sixty-four pieces of 20×20×5 mm^3^ AISI 5130 steel were case hardened using muffle furnace (2500 °C max capacity) at respective temperatures and time of 950, 1000, 1050, 1100 °C and 60, 90, 120, 180 min.
Data source location	Covenant University. Ota. Ogun-State. Nigeria.
Data accessibility	Data are available within this article

**Value of the data**

• The dataset for the surface, interface and core hardness can be used to predict optimum carburizing temperature of the treated sample.
• Data on the holding time of bi- nano additives treatment of AISI 5130 could be used to determine additives concentration in mix match ratio.
• The data could be used to predict critical temperatures for the optimum carburizing index parameters.
• Also, the dataset obtained could be used in studying the heat treatment behaviour during carburization.
• Data acquired for weight loss, wear volume and rate could be used to check the rate of the case-carbon index during heat treatment.

## 1 Data

The research work engaged the used of eggshell as energizer with palm kernel shell and coconut shell as carbon additives. The method can be applied as a part of the heat treatment of steel materials intended to be used to produce industrial tools and devices. Elemental compositional analyses were checked as indicated in Table 1. The dataset of the hardness values that was shown in Table 2 represents the sampling test template. Weight loss and wear volume data were recorded in Table 3 while Table 4 showed the summary of the surface, interface and core hardness of the treated sample. The values for the wear rate and average hardness of each sample were recorded in Table 5.

**Table 1:** Elemental composition of AISI 5130.

Elements	Composition (%)
Fe	97.377
C	0.350
Mn	0.950
P	0.050
S	0.045
Cr	1.000
Al	0.022
Mo	0.206

**Table 2:** Sampling test template.

Carburizing temperature (°C)	Holding time (min)	Wear rate test	Hardness test
950	60	1A	1B
950	90	2A	2B
950	120	3A	3B
950	180	4A	4B
1000	60	5A	5B
1000	90	6A	6B
1000	120	7A	7B
1000	180	8A	8B
1050	60	9A	9B
1050	90	10A	10B
1050	120	11A	11B
1050	180	12A	12B
1100	60	13A	13B
1100	90	14A	14B
1100	120	15A	15B
1100	180	16A	16B
	Control	17A	17B

**Table 3:** Results for the weight loss and wear volume.

Sample	Weight loss (g)	Wear volume (cm^3^)
1A	0.22	0.028
2A	0.21	0.027
3A	0.20	0.026
4A	0.19	0.024
5A	0.18	0.023
6A	0.17	0.022
7A	0.16	0.020
8A	0.11	0.009
9A	0.13	0.018
10A	0.12	0.017
11A	0.11	0.015
12A	0.10	0.014
13A	0.09	0.012
14A	0.10	0.013
15A	0.11	0.014
16A	0.12	0.015
Control	0.15	0.019

**Table 4:** Summary of results for surface, interface and core hardness.

Sample	Surface hardness (HV)	Interface hardness (HV)	Core hardness (HV)
1B	72.3	81.1	87.0
2B	89.7	94.2	93.1
3B	110.2	106.7	101.6
4B	119.6	118.3	105.2
5B	94.7	97.3	107.1
6B	120.1	96.1	97.1
7B	140.3	93.8	98.2
8B	146.9	91.7	99.8
9B	107.1	103.2	103.8
10B	113.0	141.0	169.1
11B	118.0	120.1	117.6
12B	124.7	129.0	118.7
13B	116.1	120.2	112.6
14B	137.6	124.2	119.2
15B	138.0	121.4	115.6
16B	140.9	122.2	117.9
Control	110.9	106.9	103.6

**Table 5:** Table of results from wear rate and hardness test.

Carburizing temperature (°C)	Holding time (min)	Wear rate (×10^−^^7^ cm^2^)	Hardness (Average) (HV)
950	60	2.97	80.13
950	90	2.82	92.33
950	120	2.68	106.17
950	180	2.53	114.37
1000	60	2.39	99.7
1000	90	2.24	104.43
1000	120	2.10	110.77
1000	180	1.95	112.80
1050	60	1.80	104.70
1050	90	1.16	141.03
1050	120	1.51	118.57
1050	180	1.37	124.13
1100	60	1.22	116.30
1100	90	1.35	127.22
1100	120	1.49	125.23
1100	180	1.62	127.11
			107.13
	Control		

## 2 Experimental design, materials and methods

Bi- nano additives consisting of pulverized palm kernel and coconut shell with eggshell as an energizer were used as raw materials [1], [2], [3], [4], [5]. Steel boxes of four sets with a dimension of 150×150×150 mm^3^ and sixty-four pieces of AISI 5130 steel (20×20×5 mm^3^) were also used during the research work. The elemental composition shown in Table 1 was first carried out with spectrometer before carburization. Sixteen boxes contained four each of AISI 5130 steel (0.35% carbon) were packed with additives mixed at varying weight ratio of 50:30:20 [6], [7], [8], [9], [10]. Four set of steel boxes contained sixty pieces of AISI 5130 steel were carburized using muffle furnace (2500 °C max capacity) at respective carburizing temperatures and time of 950, 1000, 1050, 1100 °C and 60, 90, 120, 180 min. The sampling template of each carburizing temperature and holding time was carefully considered as indicated in Table 2 in order to obtain the optimum temperature and time at regular interval [11], [12], [13], [14], [15], [16], [17]. Weight loss and wear volume of each sample was investigated using Rotopol-V during machining as recorded in Table 3. The test for core, interface and surface hardness of the samples were done using hardness tester shown in Table 4 [18], [19]. The values for the wear rate and average hardness of each sample were carried out and all were indicated in Table 5 [20].

## Figures and Tables

**Figure:**